# Effects of post-exercise hot and cold-water immersion on performance recovery and physiological markers in athletes: a systematic review

**DOI:** 10.3389/fspor.2026.1794029

**Published:** 2026-07-03

**Authors:** Juan Bustos Carvajal, Elkin Pérez Hurtado, Mónica Bustos Carvajal, Diego Mauricio Celis Villamizar, Florencio Arias Coronel

**Affiliations:** 1 Faculty of Education, University of Pamplona, Pamplona, Colombia; 2 Research Group in Parasitic, Tropical and Infectious Diseases (GIEPATI), University of Pamplona, Pamplona, Colombia; 3 Faculty of Health, Universidad Santiago de Cali, Cali, Colombia

**Keywords:** athletic performance, cold water immersion, exercise-induced muscle damage, hot water immersion, hydrotherapy, muscle fatigue, muscle recovery

## Abstract

Cold water immersion (CWI) and hot water immersion (HWI) are widely used recovery strategies in sports, yet their comparative efficacy remains unclear. This systematic review evaluates the effects of post-exercise CWI and HWI on performance recovery and physiological markers in athletes.

**Methods:**

Following PRISMA guidelines, a systematic search of Scopus, Web of Science, PubMed, and other databases was conducted up to December 2024. Randomized controlled trials and crossover studies comparing CWI, HWI, and control conditions in athletes or physically active individuals were included. Outcomes included perceptual recovery (DOMS, fatigue), biochemical markers (CK, cytokines), hormonal responses, and performance metrics.

**Results:**

Eight studies were included. Neither CWI nor HWI consistently reduced DOMS or improved perceived recovery compared to control. However, HWI significantly improved sleep quality and acute anaerobic power, while CWI impaired immediate power output. Biochemically, chronic HWI reduced CK and increased testosterone, whereas CWI elevated HSP-72. Hormonal and inflammatory responses were modest, with no significant changes in cortisol or testosterone to cortisol ratio.

**Conclusions:**

The effects of water immersion are temperature and context dependent. HWI may benefit sleep and anaerobic performance, while CWI may hinder immediate power and induce cellular stress adaptations. Recovery strategy selection should align with specific athletic goals.

**Systematic Review Registration:**

https://www.crd.york.ac.uk/prospero/display_record.php?ID=CRD420251156387, CRD420251156387.

## 1 Introduction

In the context of sports and exercise sciences, the optimization of athletic performance is a multifactorial goal that depends on a precise balance between the application of training stimuli and the implementation of appropriate recovery strategies. Effective recovery is a critical pillar not only for restoring physiological homeostasis and facilitating chronic adaptations to training, but also for minimizing the risk of injury, preventing overtraining, and ensuring long term adherence to exercise programs (1–3). When rest and recovery periods are insufficient, athletes may experience cumulative fatigue that compromises performance and increases susceptibility to injury (4).

High-intensity physical exercise, particularly that which predominantly involves an eccentric component, induces a phenomenon known as exercise-induced muscle damage (EIMD) (5, 6). This process is characterized at the ultrastructural level by microtrauma within muscle fibers, including sarcomere disruption and alterations in sarcolemmal integrity (6, 7). Physiologically, such damage triggers an acute inflammatory response necessary for tissue repair and remodeling (5). The clinical manifestations of EIMD include a reduction in the muscle's functional capacity, such as loss of strength and power, and the onset of delayed-onset muscle soreness (DOMS)—a sensation of discomfort and stiffness that typically peaks between 24 and 72 h after exercise (7). At the systemic level, EIMD is evidenced by the release of intramuscular proteins into the bloodstream, such as creatine kinase (CK) and myoglobin, which are widely employed as indirect biochemical markers of the extent of muscle damage (8–10).

To speed up the restoration process and mitigate the symptoms of EIMD, athletes and coaches turn to a variety of recovery strategies. Among the most widespread are active rest, massage therapy, the use of compression garments, nutritional interventions and, notably, therapies based on immersion in water (11, 12). The latter have gained remarkable popularity due to their relative ease of application and perceived benefits.

Within hydrotherapies, cold water immersion (CWI) and hot water immersion (HWI) are two of the most researched modalities, although their physiological foundations are opposite. CWI, which involves immersion in water at temperatures generally below 20 °C, is postulated to exert its effects through cold induced vasoconstriction (13). This narrowing of blood vessels can reduce edema, attenuate the inflammatory response by limiting the migration of immune cells to the injured tissue, and decrease the perception of pain by reducing nerve conduction velocity (14, 15). In contrast, HWI relies on heat induced vasodilation, which increases muscle blood flow. Theoretically, this increase in perfusion could facilitate the elimination of metabolic byproducts generated during exercise and improve the supply of oxygen and nutrients necessary for tissue repair processes (16).

Despite the existence of plausible mechanisms of action for both interventions, the current scientific literature presents conflicting results and a lack of consensus on their comparative efficacy. Some studies have suggested that CWI is superior for reducing DOMS and accelerating recovery of muscle function (11), while other research has found no significant differences between CWI, HWI, and passive recovery (25, 26). This inconsistency in the findings can be attributed, in part, to the large heterogeneity in the research protocols used (different temperatures, dive durations, and types of previous exercise).

Therefore, the objective of this systematic review is to comprehensively evaluate the effectiveness of cold-water immersion compared to hot water immersion as a post-exercise recovery strategy. The research question addressed, formulated under the PICO framework, is: In athletes and physically active individuals (P), how does cold water immersion (I) compare to hot water immersion (C) in terms of functional recovery, muscle pain perception, and biochemical markers of damage (O)?

## 2 Materials and methods

This study was conducted following the recommendations of the Cochrane Collaboration and the PRISMA statement. The protocol was registered in the international prospective registry of systematic reviews (Prospero): CRD420251156387.

The review focused on studies that met the following PICOS criteria: (P) Physically active adults or athletes undergoing exercise-induced fatigue or muscle damage. (I) Immersion in cold water (CWI) as a post-exercise recovery strategy. (C) Immersion in hot water (HWI) and/or a control condition (CON/Passive recovery). (O) Functional recovery (strength, power), biochemical markers (CK, IL-6), and perceptual recovery (DOMS, fatigue). (S): Randomized controlled trials (RCTs), randomized crossover trials, and quasi-experimental studies with a control group.

### 2.1 Criteria

#### 2.1.1 Inclusion criteria

Studies that meet the following criteria will be included in this review:

Study Design (S): Inclusion was restricted to Randomized Controlled Trials (RCTs), randomized crossover trials, and quasi-experimental studies (pre-post designs with a control group) that investigated the acute or chronic effects of water immersion on recovery markers.

Population: Athletes of any discipline and competitive level, or physically active individuals of young ages (who regularly participate in structured training programs and who have been subjected to an exercise protocol designed to induce fatigue or muscle damage.

Intervention: The primary intervention should be the application of one or more forms of aquatic or thermal therapy for the purpose of facilitating post-exertional recovery. This includes, but is not limited to: hydrotherapy, cold water immersion (CWI), hot water immersion, contrast water therapy (CWT), cryotherapy (whole body or local), and heat therapy.

Comparator: Studies should include a control group or a comparison condition, such as passive recovery (total rest), no intervention, or a placebo intervention.

Results: The study must measure and report at least one of the following primary or secondary outcomes:

Primary: Late onset muscle pain (DOMS), markers of exercise induced muscle damage (EIMD) (e.g., Creatine Kinase—CK), and perception of muscle fatigue.

Secondary: Performance in subsequent exercise (e.g., jump, sprint, strength), inflammatory markers, and joint range of motion (ROM).

Language: Articles published in Spanish or English.

The ethical approval and adherence to the Declaration of Helsinki of all primary studies were verified during the selection process.

#### 2.1.2 Exclusion criteria

Studies that meet one or more of the following conditions will be excluded from this review:

Type of Study: Systematic reviews, meta-analyses, letters to the editor, editorials, conference abstracts, single case studies, books or book chapters.

Population: Studies conducted exclusively in sedentary populations, patients with chronic non sport related pathologies (e.g., fibromyalgia, arthritis, circulatory diseases), or in animal models.

Procedure: Studies in which aquatic or thermal therapies are applied chronically (as part of training) and not as an acute intervention for post-exercise recovery.

Interventions that combine therapies of interest with other active treatments (e.g., massage, compression, supplementation) if the effects of aquatic/thermal therapy cannot be independently analyzed.

Therapies applied before exercise in order to improve performance (warm-up), rather than after for recovery.

Results: Studies that do not report any of the outcomes of interest defined in the inclusion criteria (e.g., studies that only measure body temperature or psychological variables such as mood without a measure of physical recovery or performance).

Availability: Items from which the full text cannot be obtained after reasonable attempts.

### 2.2 Factor to evaluate

#### 2.2.1 Primary result

Sources of information: The bibliographic search was carried out in the databases: Scopus, Science Direct, WebofScience, Sportdiscuss and Sage Journal, controlled clinical trials, case-control studies, cohorts and cross-sectional studies were included from the beginning to December 2024, to ensure the saturation of the literature, references of relevant articles identified through references, congresses, thesis databases, Open Grey, Google Scholar and Clinicaltrials.gov were scanned.

The search was performed using DeCS/Mesh terms and related words, the different combinations with the Boolean operators were used. No language or time restrictions were imposed. (Appendix A). Search strings were adapted to the specific syntax and Boolean operators of each database to ensure conceptual equivalence. Filters in Scopus (e.g., excluding “notes” or “reviews”) were applied to strictly align with the inclusion criteria established in the PICOS framework, ensuring that only primary research articles were retrieved across all platforms.

#### 2.2.2 Study selection and data collection process

To ensure transparency and reproducibility, a reference management software (Zotero) was used to automatically remove duplicates. Two authors (EP-H and JSB-C) independently screened the titles and abstracts of all identified records using the Rayyan online tool. Subsequently, the full texts of potentially eligible studies were retrieved and assessed by the same two reviewers to confirm they met the PICOS inclusion criteria. Any discrepancies were resolved through discussion or, if necessary, by consulting a third senior reviewer (FA-C). The search was last updated in December 2024. Data extraction followed a standardized protocol and included the following: Bibliographic characteristics: study design, location, authors, and title. Methodological aspects: objectives, sample size. Outcome variables: definitions, reported results and measures of effect. When numerical data were not explicitly reported in tables or text, values were visually estimated from the original figures using digital calipers or scale projection. An inherent imprecision of approximately ±5% was assumed for these extracted data points.

#### 2.2.3 Risk of bias assessment (quality)

The methodological quality of the studies and the risk of bias were assessed using specific instruments for clinical trials. First, the PEDro scale was applied, which assesses fundamental aspects such as randomization, blinding and the presentation of results. The included studies scored between 6 and 7 on this scale (Table 1). Additionally, the Cochrane tool for randomised trials (RoB 2) was used in order to examine in more detail the risk of bias in the studies analysed (Table 2).

**Table 1:** PeDro scale.

Estudio	1	2	3	4	5	6	7	8	9	10	11	PEDro score
1. Horgan et al. (17) acute Inflammatory, anthropometric, and perceptual (muscle soreness) effects of postresistance exercise water immersion in junior international and subelite male volleyball athletes	✔	✔	✘	✔	✘	✘	✘	✔	✔	✔	✔	6 de 10
2. Colomer et al. (18) monitoring jump performance and DOMS following resistance training and hydrotherapy	✔	✔	✘	✔	✘	✘	✘	✔	✔	✔	✔	6 de 10
3. Wellauer et al. (19) no acceleration of recovery from exercise-induced muscle damage after cold or hot water immersion in women	✔	✔	✔	✔	✘	✘	✘	✔	✔	✔	✔	7 de 10
4. Menzies et al. (20) post-exercise hot or cold-water immersion does not alter perception of effort or neuroendocrine responses	✔	✔	✘	✔	✘	✘	✘	✔	✔	✔	✔	6 de 10
5. Gustafsson et al. (21) cold- and hot-water immersion are not more effective than placebo in soccer players	✔	✔	✘	✔	✘	✘	✔	✔	✔	✔	✔	7 de 10
6. Horgan et al. (22) acute performance, daily well-being, and hormone responses to water immersion in volleyball athletes	✔	✔	✘	✔	✘	✘	✘	✔	✔	✔	✔	6 de 10
7. Coertjens et al. (23) energetic responses of head-out water immersion at different temperatures during post-exercise recovery	✔	✔	✘	✔	✘	✘	✘	✔	✔	✔	✔	6 de 10
8. Horgan et al. (24) effect of repeated post-resistance exercise cold or hot water immersion on inflammatory responses in rugby players	✔	✔	✘	✔	✘	✘	✘	✔	✔	✔	✔	6 de 10

PEDro scale criteria: (1) choice criteria were specified (*this item is not used to calculate the PEDro score). (2) Subjects were randomly assigned to groups (in a crossover study, subjects were randomly assigned in an order in which they received treatments). (3) Assignment was hidden. (4) Groups were similar at baseline with respect to the most important prognostic indicators. (5) There was blinding of all subjects. (6) There was blinding of all therapists who administered therapy. (7) There was blinding of all assessors who measured at least one key outcome. (8) Measures of at least one key outcome were obtained from more than 85% of subjects initially assigned to groups. (9) All subjects for whom outcome measures were available received the treatment or control condition as assigned or, when this was not the case, data on at least one key outcome were analyzed by “intention-to-treat.” (10) Results of statistical comparisons between groups are reported for at least one key outcome. (11) The study provides point measures and measures of variability for at least one key outcome.

**Table 2:** Tool (RoB 2).

Study	D1: randomization process	D2: Deviations from intended interventions	D3: missing outcome data	D4: measurement of the outcome	D5: Selection of the reported result	Overall risk of bias
Horgan et al. (22)	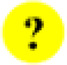	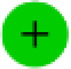	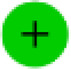	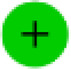	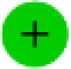	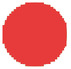
Colomer et al. (18)	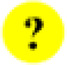	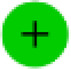	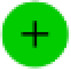	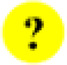	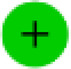	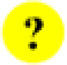
Horgan et al., (22)	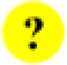	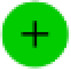	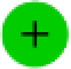	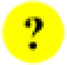	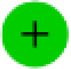	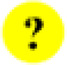
Coertjens et al. (23)	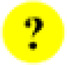	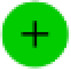	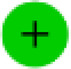	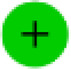	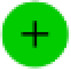	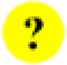
Horgan et al., (24)	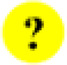	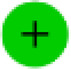	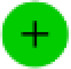	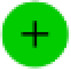	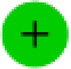	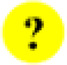
Wellauer et al. (19)	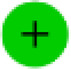	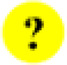	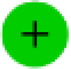	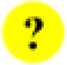	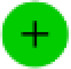	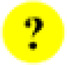
Menzies et al. (20)	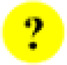	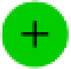	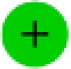	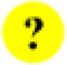	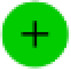	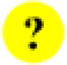
Gustafsson et al. (21)	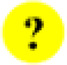	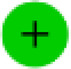	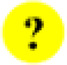	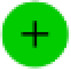	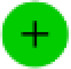	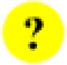

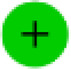
, Low risk of bias; 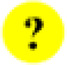, some concerns; 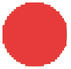, high risk of bias.

### 2.3 Data synthesis and assessment of heterogeneity

A narrative synthesis approach was adopted for this review. A meta-analysis was not performed due to the high methodological heterogeneity identified among the included studies, including variations in water temperature (CWI: 8–15 °C; HWI: 38–42 °C), immersion duration (5–20 min), and the nature of the preceding exercise stimulus (e.g., resistance training vs. endurance sports). These discrepancies in protocols precluded a meaningful quantitative pooling of data.

### 2.4 Definition of outcome variables

To ensure a comprehensive evaluation of post-exercise recovery, the following primary and secondary variables were analyzed, categorized into three distinct physiological dimensions:

Perceptual Recovery Markers: Delayed Onset Muscle Soreness (DOMS): Quantified using Visual Analogue Scales (VAS) to assess the subjective sensation of pain and stiffness that typically peaks 24–72 h post-exercise.

Perceived Fatigue and Well-being: Evaluated through psychometric tools (e.g., ARSS or Likert scales) to reflect the athlete's subjective state of readiness and overall systemic tiredness.

Sleep Quality: Assessed to determine the impact of thermal therapy on nocturnal restoration and central nervous system recovery.

Biochemical and Hormonal Markers: Creatine Kinase (CK): Measured as the gold-standard indirect marker of sarcolemmal disruption and exercise-induced muscle damage (EIMD).

Inflammatory Cytokines (IL-6, IL-1ra): Analyzed to evaluate the magnitude of the acute inflammatory response and the subsequent anti-inflammatory modulation.

Testosterone and Cortisol: Assessed to determine the anabolic-to-catabolic balance (T:C ratio) and the neuroendocrine stress response to the training-recovery cycle.

Heat Shock Proteins (HSP-72): Used as a cellular-level marker of thermal stress and proteostatic adaptation.

Performance and Physiological Markers: Neuromuscular Function: Evaluated via Countermovement Jump (CMJ) height and Maximal Voluntary Isometric Contraction (MVIC) to track the recovery of explosive and absolute strength.

Anaerobic Potency: Measured through the Wingate Test (WAnT) to assess the acute effects of water temperature on muscle enzyme kinetics and peak power output.

Systemic Physiological Responses: Including heart rate (HR), core/skin temperature, and femoral blood flow, to elucidate the underlying thermoregulatory and cardiovascular mechanisms of hydrotherapy.

## 3 Results

**Figure 1:**
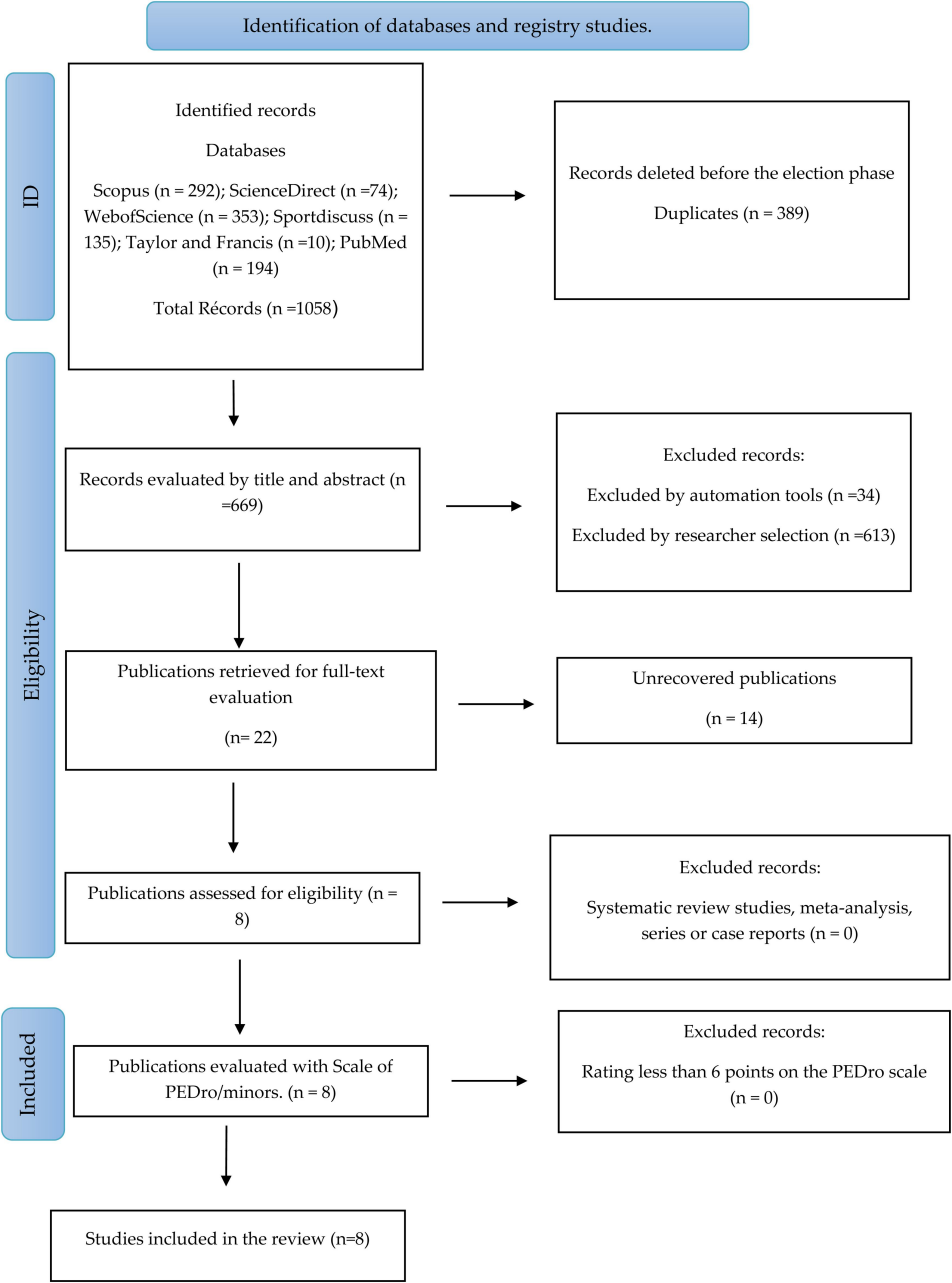
Prism diagram.

A total of 1,058 records were initially identified through database searches. After removing 389 duplicates, 669 records were screened by title and abstract. Of these, 647 were excluded based on inclusion/exclusion criteria. From the 22 publications retrieved for full-text evaluation, 14 were unrecovered or did not meet eligibility, resulting in 8 studies that met the criteria and were included in this systematic review (Figure 1).

A total of eight studies were included for analysis. Methodologically, all the articles corresponded to randomized controlled clinical trials, with a notable predominance of the crossover design, used in six of the eight investigations. The geographical distribution of the evidence was mainly concentrated in Oceania, with four studies carried out in Australia, followed by Europe, with three studies (from Switzerland, Belgium, the United Kingdom and Sweden), and one study from the American continent (Brazil). All articles were published in English in a recent period, between 2017 and 2025 (Table 3).

**Table 3:** Bibliographic characteristics.

#	Authors	Qualification	Study type	Country	Language	Year	Aim
1	Horgan et al. (17)	18 international junior and sub-elite male volleyball athletes.	Randomized crossover clinical trial (RCT-crossover).	Australia	English	2022	Investigate the acute inflammatory, anthropometric, and perceptual effects of post-resistance exercise water immersion.
2	Colomer et al. (18)	19 male volleyball players at the sub-elite level. (24.1 ± 5.5 years)	ECA-crossover.	Australia	English	2017	To assess the acute effects of hydrotherapy on recovery of jump performance and delayed onset muscle soreness (DOMS) after resistance training.
3	Wellauer et al. (19)	30 healthy and recreationally active women. (23.3 ± 2.9 years)	Parallel group RCTs.	(Suiza-Bélgica).	English	2025	To compare the effects of cold water immersion (CWI) and hot water immersion (HWI) on muscle recovery after muscle damage exercise in women.
4	Menzies et al. (20)	14 trained endurance runners and triathletes (men and women). (28 ± 7 years)	ECA-crossover.	United Kingdom	English	2024	Investigate whether post-exercise water immersion (cold or hot) alters the perception of exertion and neuroendocrine responses in a subsequent exercise on the same day.
5	Gustafsson et al. (21)	40 national male footballers (15–19 years old).	RCT with CON group.	Sweden	English	2025	(1) Compare CWI, HWI and placebo at recovery. 2) Evaluate impact of repeated use over 15 weeks.
6	Horgan et al. (22)	18 international junior and sub-elite male volleyball athletes. (19.9 ± 3.4 years)	ECA-crossover.	Australia	English	2023	Investigate acute effects of 3 water immersion strategies on performance, well-being and hormonal responses after endurance exercise.
7	Coertjens et al. (23)	21 trained male cyclists. (26.9 ± 5.8 years)	ECA-crossover.	Brazil	English	2023	Investigate energy responses of immersion in water at different temperatures during post-exercise recovery and its effect on subsequent anaerobic potency.
8	Horgan et al. (24)	18 male academy rugby players (Super Rugby). (19.9 ± 1.5 years)	ECA-crossover (pre-post by blocks).	Australia	English	2024	Investigate the effects of repeated immersion (cold or hot) post-resistance exercise on seasonal inflammatory and hormonal responses.

### 3.1 Characteristics of excluded studies

Excluded items showed no connection to the result of interest. In addition, letters to the editor, systematic/narrative reviews, medication intervention protocols were dispensed with.

### 3.2 Methodological quality

The average score on the PEDro scale for the studies analyzed, detailed in Table 2, was 5 points with methodological quality studies, observing that the most frequent omissions were observed in the blinding of all therapists who administered the therapy and in the blinding of all evaluators who measured at least one key outcome.

### 3.3 Characteristics of the participants

Samples ranged from 14 to 40 participants. Subjects’ age ranged from adolescent athletes (15–19 years) to young adults, with most research focusing on the 20–30 age range. Regarding the distribution by sex, a clear predominance of the male sex was observed, with six of the eight studies including exclusively men, one study carried out only with women and one with a mixed sample. The population studied was diverse in terms of sports discipline, including team sports (volleyball, football, rugby) and endurance sports (cycling, running); one study was conducted with recreationally active participants instead of competitive athletes. It is noteworthy that all studies with athletes focused on a high-level population, described as “trained”, “sub-elite”, “elite” or “academy”, which ensures the applicability of the findings to the field of sports performance (Table 4).

**Table 4:** Results of perceptual and subjective measures (DOMS, fatigue, sleep quality, RPE, general recovery).

Study	Cluster (group)	Extent (parameter)	Initial value	Final value	Difference/Tracking	*p* value
Colomer et al. (18)	CON	DOMS (mm shift)^a^	(Baseline)	∼ + 25 mm (Peak 24–48 h)	Significantly increase	Time effect: *p* < 0.03 Intervention: NS^b^
	CWI (cold water)	DOMS (mm shift)^a^	(Baseline)	∼ + 28 mm (Peak 24–48 h)	Significantly increase	—
	CWT (contrast)	DOMS (mm shift)^a^	(Baseline)	∼ + 30 mm (Peak 24–48 h)	Significantly increase	—
	HWI (hot water)	DOMS (mm shift)^a^	(Baseline)	∼ + 30 mm (Peak 24–48 h)	Significantly increase	—
Wellauer et al. (19)	CON	DOMS (VAS 0–10 cm)	1.0 cm	∼ 6.5 cm (24 h Peak)	Significantly increase	Time effect: *p* < 0.001 Intervention: NS^b^ (*p* = 0.752)
	CWI (cold water)	DOMS (VAS 0–10 cm)	1.0 cm	∼ 6.5 cm (24 h Peak)	Significantly increase	—
	HWI (hot water)	DOMS (VAS 0–10 cm)	1.0 cm	∼7.0 cm (24 h Peak)	Significantly increase	—
Menzies et al. (20)	CON	General recovery (ARSS)	4.0 ± 0.8	3.4 ± 0.7	−0.6	Time effect: *p* = 0.0003 Intervention: NS^b^ (*p* = 0.31)
	CWI (cold water)	General recovery (ARSS)	3.7 ± 0.6*	3.3 ± 0.6	−0.4	—
	HWI (hot water)	General recovery (ARSS)	3.9 ± 0.6*	3.4 ± 0.6*	−0.5	—
	CON	Perception of effort (RPE 6–20)	15.1 ± 2.2	15.2 ± 2.3	No change	Intervention: NS^b^ (*p* = 0.68)
	CWI (cold water)	Perception of effort (RPE 6–20)	14.9 ± 2.4	14.8 ± 2.6	No change	—
	HWI (hot water)	Perception of effort (RPE 6–20)	15.0 ± 2.3	15.2 ± 2.4	No change	—
Gustafsson et al. (21)	CON	Perception of effort (RPE 1–10)	∼6.5	∼ 7.5 (Peak 21 h)	Significantly increase	Time effect: *p* < 0.05 Intervention: NS^b^ (*p* = 0.45)
	CWI (cold water)	Perception of effort (RPE 1–10)	∼6.5	∼ 7.5 (Peak 21 h)	Significantly increase	—
	HWI (hot water)	Perception of effort (RPE 1–10)	∼6.5	∼7.6 (Peak 21 h)	Significantly increase	—
Horgan et al. (22)	HWI vs. CON	Sleep quality (VAS 0–100 mm)^c^	N/A	N/A	HWI significantly better than WITH	*p* < 0.001
	HWI vs. CWI	Sleep quality (VAS 0–100 mm)^c^	N/A	N/A	HWI significantly better than WITH	*p* = 0.017
	HWI vs. CWT	Fatigue (VAS 0–100 mm)^c^	N/A	N/A	HWI significantly less than CWT	*p* = 0.050

Coertjens et al. (23): The exclusion of this study in the table is maintained due to its qualitative evaluation of preference, which does not fit into the quantitative format. ARSS, Acute Recovery and Stress Scale; CON, control group; CWI, cold water immersion; CWT, contrast water therapy; DOMS, late onset muscle pain; HWI, hot water immersion; NS, not significant; RPE, effort perception scale; VAS, visual analogue scale.

* Significant difference compared to the initial value (*p* < 0.05).

a DOMS (mm change): Initial and final values are visual estimates extracted from the original study figures, as exact numerical data were not provided in text or tables. These estimates carry an inherent degree of imprecision (approx. ± 5% based on axis scale resolution).

b NS (not significant): The *p*-value of the intervention indicates that there were no statistically significant differences between the groups. This is a key finding: although athletes felt worse after exercise (time effect), the recovery strategy did not significantly change that outcome compared to the others.

c Direct Comparison: The study by Horgan et al. (22) does not report pre-post data for these variables, but direct comparisons of the effect of each treatment. The table reflects which group was higher and the *p*-value of that comparison. N/A (not applicable) is used because the table format does not fit this type of analysis.

### 3.4 Most evaluated parameters

The parameters evaluated in the included studies were grouped into three main categories: perceptual and subjective measures, blood biochemical markers, and objective neuromuscular performance. Perceptual measures, present in most investigations, included assessment of delayed onset muscle soreness (DOMS), fatigue, sleep quality, and overall perception of recovery, frequently quantified using visual analogue scales (VAS).

In the biochemical realm, muscle damage was a key parameter, systematically assessed through serum Creatine Kinase (CK) concentration. Additionally, markers of the inflammatory response, such as various cytokines (e.g., IL-6, IL-1ra), and hormonal responses (testosterone and cortisol) were analyzed.

Neuromuscular performance was quantified primarily through countermovement jump height (CMJ) and maximal voluntary isometric force (MVIC), representing objective measures of muscle function. More specific parameters of sports performance, such as sprint speed over short distances and anaerobic power (measured with the Wingate test), were evaluated less frequently. A subgroup of studies also investigated acute systemic physiological responses, such as body temperature, heart rate, blood flow, and oxygen consumption (VO_2_), in order to elucidate the underlying mechanisms of interventions.

### 3.5 Results of the evaluated parameters

Analysis of perceptual and subjective measures (Table 4) reveals a predominant and consistent finding in most studies: hydrotherapy interventions, regardless of temperature, were not shown to be superior to control or placebo conditions to improve athletes' perceived recovery.

Specifically, with regard to late onset muscle pain (DOMS), the studies by Colomer et al. (18) and Wellauer et al. (19) showed that, although pain increased significantly in all groups as a response to exercise, neither cold water immersion (CWI) nor hot water immersion (HWI) managed to attenuate this response more effectively than the control group. Similarly, perceived exertion (RPE) and overall recovery followed an analogous trajectory; work by Menzies et al. (20) and Gustafsson et al. (21) concluded that hydrotherapy interventions did not reduce perceived exertion in a subsequent exercise session or improve feelings of recovery compared to control or a credible placebo.

In contrast to this general trend, the study by Horgan et al. (22) identified specific and selective benefits. In this research, hot water immersion (HWI) was the only modality that demonstrated a statistically significant improvement in sleep quality (compared to CWI and control) and a reduction in fatigue (compared to contrast therapy). This discrepancy could be attributed to the type of previous exercise (resistance training in the Horgan study vs. endurance/team sports in the others), suggesting that the perceptual benefits of HWI could be more pronounced after strength stimuli.

Taken together, these findings suggest that, for the mitigation of general muscle pain and the perception of exertion, the effects of hydrotherapy are, at best, equivalent to those of passive rest or placebo. However, hot water immersion could offer a specific and relevant benefit to optimize sleep quality and reduce fatigue, especially in strength training contexts.

The analysis of blood markers (Table 5) presents a complex and sometimes contradictory picture, suggesting that the effects of hydrotherapy depend on both the specific marker and the chronicity of the intervention (acute vs. repeated).

**Table 5:** Blood markers (muscle damage and inflammation) (creatine kinase—CK, cytokines).

Study	Cluster (group)	Extent (parameter)	Initial value	Final value	Difference/Tracking	*p* value
Horgan et al. (22)^a^	CWI vs. WITH	Creatine kinase (CK)	N/A	N/A	Significant reduction of CK vs. CON	*p* = 0.007
	CWT vs. WITH	Creatine kinase (CK)	N/A	N/A	Significant reduction of CK vs. CON	*p* = 0.006
	HWI vs. CON	Creatine kinase (CK)	N/A	N/A	Significant reduction of CK vs. CON	*p* < 0.001
	CWT vs. HWI	Interleukin-6 (IL-6)	N/A	N/A	Highest IL-6 after CWT (not significant, *g* = 0.56)	NS^b^
Wellauer et al. (19)^a^	CON	Creatine kinase (CK) (U/L)	119.8 ± 50.2	700 (24 h peak)	Significant post-exercise increase	Time: *p* < 0.001
	CWI (cold water)	Creatine kinase (CK) (U/L)	111.0 ± 41.5	600 (24 h peak)	Significant increase, no diff. vs. CON	Interv.: NS^b^
	HWI (hot water)	Creatine kinase (CK) (U/L)	214.3 ± 180.9	850 (24 h peak)	Significant increase, higher than AT 24H	vs. CON (24 h): *p* = 0.046
Menzies et al. (20)^a^	CON	Interleukin-6 (IL-6) (pg/mL)	5	∼2.0 (Post-EX peak)	Post-e.g., increase, no significant diff.	Interv.: NS^b^
	CWI (cold water)	Interleukin-6 (IL-6) (pg/mL)	5	∼2.2 (Post-EX peak)	Post-e.g., increase, no significant diff.	NS^b^
	HWI (hot water)	Interleukin-6 (IL-6) (pg/mL)	5	∼2.0 (Post-EX peak)	Post-e.g., increase, no significant diff.	NS^b^
Horgan et al. (24)^c^	CWI vs. WITH	Creatine kinase (CK)	N/A	N/A	Trivial but significant reduction after 4 weeks	*p* = 0.025
	HWI vs. CON	Creatine kinase (CK)	N/A	N/A	Moderate and significant reduction after 4 weeks	*p* < 0.001
	HWI vs. CON	Interleukin-1ra (IL-1ra) (Δ)	N/A	N/A	Moderate (anti-inflammatory) reduction after 4 weeks	*p* = 0.013
	CWI vs. HWI	Heat shock protein (HSP-72) (Δ)	N/A	N/A	Small increase of HSP-72 in CWI after 4 weeks	*p* = 0.004

N/A (not applicable): used when studies present results as direct comparisons between groups (e.g., “group A was significantly larger than group B”) rather than reporting initial and final numerical values. Excluded studies: the studies of Colomer et al. (18), Gustafsson et al. (21) and Coertjens et al. (23) did not measure CK or inflammatory cytokines and are therefore not included in this table. CK, creatine kinase; CON, control group; CWI, cold water immersion; CWT, contrast water therapy; *g*, *g* Hedges (effect size); HWI, hot water immersion; HSP-72, heat shock protein 72; IL-1ra, interleukin-1 receptor antagonist; IL-6, interleukin-6; N/A, not applicable; NS, not significant; *p*, *p*-value; U/L, units per liter; Δ, change from baseline.

a Acute studies: the results of Horgan et al. (22), Wellauer et al. (19), and Menzies et al. (20) reflect the response to a single session of exercise and intervention.

b NS (not significant): indicates that there was no statistically significant difference between the intervention groups for that specific parameter, even though the parameter changed over time due to exercise. In the case of Horgan et al. (17), the *p*-value for IL-6 was not significant, but the effect size (*g* = 0.56) suggests a mean difference.

c Chronic study: the results of Horgan et al. (24) reflect the cumulative effects of repeated interventions over 4 weeks. Therefore, the result is presented as a change (Δ) in baseline levels.

As for the main indicator of muscle damage, Creatine Kinase (CK), the acute effects seem inconsistent. On the one hand, Horgan et al. (17) reported that all immersion modalities (CWI, CWT, and HWI) significantly reduced CK levels compared to passive control. In direct contrast, the study by Wellauer et al. (19) found no such benefit for CWI and indeed observed that HWI resulted in significantly higher CK levels than control at 24 h post-exercise. This discrepancy could be attributed to differences in the exercise protocol (force vs. jump-induced injury), the population studied (men vs. women), or the timing of the measurement. However, when assessing chronic effects, the evidence aligns: Horgan et al. (24) showed that both CWI (trivial reduction) and HWI (moderate reduction) attenuated CK levels after 4 weeks of repeated application, with HWI being more effective.

The behavior of inflammatory cytokines was equally nuanced. Generally, exercise was the main stimulus for the increase of cytokines such as IL-6, without hydrotherapy interventions significantly modifying this acute response (20). However, specific effects were observed: Horgan et al. (17) found an increase in IL-6 after CWT compared to HWI. Most notably, the chronic study by Horgan et al. (24) revealed that repeated HWI decreased baseline levels of the anti-inflammatory cytokine IL-1ra, a finding that could be linked to the lower burden of chronic muscle damage.

Finally, a relevant mechanistic finding came from the chronic study, where repeated CWI induced an increase in Heat Shock Protein (HSP-72). This suggests a cellular adaptive response to cold heat stress, which was not observed with hot immersion.

Regarding specific high-intensity tasks (Table 6), it reveals that the effects of hydrotherapy are highly dependent on both the type of task evaluated (sprint vs. power) and the timing of the measurement (long-term recovery vs. immediate performance).

**Table 6:** Performance on specific tasks (sprint, power) (speed, anaerobic power).

Study	Cluster (group)	Extent (parameter)	Initial value	Final value	Difference/tracking	*p* value
Gustafsson et al. (21)	CON	Sprint speed (20 m) (s)^a^	Pre-match	Did not return to baseline (21–45 h post)	Sprint ↑ time (∼1.5% post-match), incomplete recovery	Time: *p* < 0.05 Interv.: NS^b^ (*p* = 0.90)
	CWI (cold water)	Sprint speed (20 m) (s)^a^	Pre-match	Did not return to baseline (21–45 h post)	Sprint ↑ time (∼1.5% post-match), incomplete recovery	NS^b^
	HWI (hot water)	Sprint speed (20 m) (s)^a^	Pre-match	Did not return to baseline (21–45 h post)	Sprint ↑ time (∼1.5% post-match), incomplete recovery	NS^b^
Coertjens et al. (23)	CON	Anaerobic average power (WAnT)^b^	WAnT₁	WAnT_2_	No significant changes (baseline)	N/A
	CWI (cold water)	Anaerobic average power (WAnT)^b^	WAnT₁	WAnT_2_	−2.4% (↓Yield)	vs. CON: *p* < 0.05 vs. HWI: *p* < 0.0001
	HWI (hot water)	Anaerobic average power (WAnT)^b^	WAnT₁	WAnT_2_	+2.2% (↑Yield)	vs. CON: *p* < 0.05 vs. CWI: *p* < 0.0001

NS (not significant): indicates that there were no statistically significant differences between the intervention groups. Excluded studies: the other 6 studies did not include direct measurements of sprint speed or maximum anaerobic power, so they are not listed in this table. CON, control group; CWI, cold water immersion; HWI, hot water immersion; m, meters; N/A, not applicable; NS, not significant; *p*, *p*-value; s, seconds; WAnT, Wingate anaerobic test.

a Sprint speed (s): measured in seconds (s), therefore an increase in numerical value means a decrease in performance (slower sprint).The study by Gustafsson et al. showed that exercise (simulated football match) worsened sprint performance, and none of the interventions (CWI, HWI or Placebo) accelerated the return to baseline values.

b Mean anaerobic power (WAnT): the study by Coertjens et al. used the Wingate test (WAnT). The table compares performance on a second test (WAnT_2_) after the recovery intervention, relative to the first test (WAnT₁). The results are very clear: hot water improved the power, while cold water significantly worsened it.

In the context of sprint speed recovery at 21–45 h post-match, the study by Gustafsson et al. (21) found no benefit. Sprint performance decreased in all groups as a result of exercise induced fatigue, and neither cold water immersion (CWI) nor hot water immersion (HWI) accelerated the return to baseline levels more effectively than the CON group. This finding suggests that, for recovery of speed in the days following competition, these hydrotherapy modalities do not offer an objective advantage.

In stark contrast, the study by Coertjens et al. (23) which evaluated anaerobic potency in a test performed minutes after the intervention, showed acute, significant and diametrically opposite effects. Hot water immersion (HWI) resulted in a 2.2% improvement in mean power, an acute potentiating effect. In contrast, cold water immersion (CWI) resulted in a 2.4% detriment in yield.

This divergence can be explained by the underlying physiological mechanisms. HWI, by increasing muscle temperature, probably optimized enzyme kinetics and contraction rate, improving power output. CWI, by reducing muscle temperature, would have slowed these processes, resulting in underperformance.

Analysis of hormonal responses (Table 7) indicates that hydrotherapy interventions, in general, have a modest effect on the hormonal axis, although specific effects of hot water immersion (HWI) are identified that could be relevant for adaptation to training.

**Table 7:** Hormonal responses (testosterone, cortisol).

Study	Cluster (group)	Extent (parameter)	Initial value	Final value	Difference/Tracking	*p* value
Menzies et al. (20)^b^	CON	Cortisol (ng/mL)	∼121 (pre-race 1)	∼92 (pre-race 2)	↓ Cortisol in all groups, with no differences between interventions	Time: *p* = 0.008 Interv.: NS^a^
	CWI (cold water)	Cortisol (ng/mL)	∼121 (pre-race 1)	∼92 (pre-race 2)	↓ Cortisol in all groups, with no differences between interventions	NS^a^
	HWI (hot water)	Cortisol (ng/mL)	∼121 (pre-race 1)	∼92 (pre-race 2)	↓ Cortisol in all groups, with no differences between interventions	NS^a^
Horgan et al. (22)^b^	HWI vs. CWT	Testosterone	N/A	N/A	Significantly higher ↑ testosterone in HWI vs. CWT	*p* = 0.038
	All the groups	Cortisol	N/A	N/A	No significant differences between interventions	INTERV.
	All the groups	Testosterone:Cortisol Ratio	N/A	N/A	No significant differences between interventions	Interv.: NS^a^
Horgan et al. (24)^c^	HWI vs. CON	Testosterone	N/A	N/A	Baseline testosterone ↑ levels after 4 weeks in HWI vs. WITH	*R*^2^: *p* = 0.012
	All the groups	Cortisol	N/A	N/A	No significant differences between interventions after 4 weeks	Interv.: NS^a^
	All the groups	Testosterone:Cortisol Ratio	N/A	N/A	No significant differences between interventions after 4 weeks	INTERV.

N/A (not applicable): used when the studies present the results as direct comparisons of the treatment effect (“group A was greater than B”) instead of reporting initial and final numerical values that allow a difference to be calculated. Excluded studies: the other 5 studies did not include hormonal measurements of testosterone or cortisol and are therefore not listed in this table. CON, control group; CWI, cold water immersion; CWT, contrast water therapy; HWI, hot water immersion; N/A, not applicable; ng/mL, nanograms per milliliter; NS, not significant; *p*, *p*-value.

a NS (not significant): indicates that there was no statistically significant difference between the intervention groups for that specific parameter. In the study by Menzies et al., the decrease in cortisol was attributed to the passage of time (circadian cycle and recovery), not to hydrotherapy.

b Acute study: the results of Menzies et al. (20) and Horgan et al. (22) reflect the hormonal response to a single session of exercise and intervention.

c Chronic study: the results of Horgan et al. (24) reflect the cumulative effects of repeated interventions over 4 weeks on resting baseline hormone levels.

The behavior of cortisol, a marker of catabolic stress, was not significantly influenced by any of the hydrotherapy modalities in the studies that evaluated it. In both the acute (20, 22) and chronic (24) contexts, fluctuations in cortisol levels were primarily attributed to the natural circadian cycle and response to exercise, with water immersion interventions not significantly altering this dynamic compared to control.

In contrast, testosterone, a key hormone for anabolic processes, was shown to be sensitive to the temperature of the dive. Acutely, Horgan et al. (22) reported that HWI induced a significantly greater increase in testosterone compared to contrast therapy (CWT). This finding was reinforced by the chronic effects observed in the study by Horgan et al. (24), where 4 weeks of repeated HWI resulted in significantly higher baseline testosterone levels compared to the control group. These results suggest that hot water immersion could promote a hormonal environment more conducive to muscle recovery and adaptation.

Despite the modifications in testosterone, the Testosterone: Cortisol (T:C) ratio, often used as an indicator of the balance between anabolism and catabolism, showed no significant changes in any of the studies.

In the context of systemic physiological responses (Table 8) reveals the most direct, pronounced and divergent effects of hydrotherapy interventions, confirming that water temperature is a potent modulator of body homeostasis. These findings explain many of the results observed in the other variables.

**Table 8:** Systemic physiological responses (temperature, heart rate, VO_2_, blood flow).

Study	Cluster (group)	Extent (parameter)	Initial value	Final value	Difference/Tracking	*p* value^a^
Wellauer et al. (19)	CON	Core Temperature ( °C)	2.	∼37.1 (30 min post)	Lightweight	HWI > CWI (*p* = 0.001)
	CWI (cold water)	Core Temperature ( °C)	2.	∼36.9 (30 min post)	↓ Significant vs. HWI & CON	
	HWI (hot water)	Core Temperature ( °C)	2.	∼38.5 (post-immersion)	Significant	
	CWI (cold water)	Skin Temperature ( °C)	$0.32	∼16.0 (post-immersion)	Drastic	vs. WITH & HWI: *p* < 0.001
Menzies et al. (20)	CON	Heart rate (bpm)	60	60	No change	Intervention: *p* < 0.0001
	CWI (cold water)	Heart rate (bpm)	60	54	↓ Below baseline rest	
	HWI (hot water)	Heart rate (bpm)	60	∼80	↑ Sustained after immersion	
	CON	Femoral blood flow (mL/min)^a^	∼200	151	Recovery to Baseline	Intervention: *p* < 0.0001
	CWI (cold water)	Femoral blood flow (mL/min)^a^	∼200	66	Flow ↓ drastic	
	HWI (hot water)	Femoral blood flow (mL/min)^a^	∼200	.203	↑ Sustained after immersion	
Gustafsson et al. (21)	CON	Heart rate (bpm)	−170	∼165 (45 h post)	PROGRESSIVE	Time: *p* < 0.01 Interv.: NS^c^
	CWI (cold water)	Heart rate (bpm)	−170	∼165 (45 h post)	PROGRESSIVE	
	HWI (hot water)	Heart rate (bpm)	−170	∼165 (45 h post)	PROGRESSIVE	
Coertjens et al. (23)	CON	HR recovery (τHR, s)^d^	N/A	64.0 ± 3.8	Recovery Baseline	Intervention: *p* < 0.0001
	CWI (cold water)	HR recovery (τHR, s)^d^	N/A	60.5 ± 3.8	Faster recovery (τ↓)	
	HWI (hot water)	HR recovery (τHR, s)^d^	N/A	94.3 ± 3.8	Slower recovery (τ↑)	
	CON	Post-exercise O_2_ consumption (EPOC) (L)	N/A	3.7 ± 0.2	Metabolic baseline	Intervention: *p* < 0.001
	CWI (cold water)	Post-exercise O_2_ consumption (EPOC) (L)	N/A	4.7 ± 0.2	↑ Significant energy expenditure	
	HWI (hot water)	Post-exercise O_2_ consumption (EPOC) (L)	N/A	4.4 ± 0.2	↑ Significant energy expenditure	

NS (not significant): Indicates that there were no statistically significant differences between the intervention groups. COPD (excessive post-exercise oxygen consumption): represents the “extra” energy expenditure the body uses to recover after exercise. A higher value means a higher metabolic cost during the recovery period. Excluded studies: the other studies did not measure these systemic physiological variables. Values preceded by (∼) were visually estimated from study graphs. These estimates carry an inherent degree of imprecision (approx. ± 5%). bpm, beats per minute; CON, control group; CWI, cold water immersion; EPOC, post-exercise excessive oxygen consumption; HWI, hot water immersion; L, liters; mL/min, milliliters per minute; NS, not significant; *p*, *p*-value; s, seconds; τHR, heart rate tau (recovery rate indicator); °C, degrees celsius.

a Acute data (post-immersion): the values of Menzies et al. represent the physiological state immediately after the intervention, showing the acute and divergent effects of each temperature.

c Long-term recovery data: Gustafsson et al. values are from a sub-maximal test 45 h later, showing that acute differences in heart rate are no longer evident.

d τHR (heart rate tau): a measure of recovery kinetics. A lower Tau (τ) value indicates a faster recovery of the heart rate towards resting levels.

Body temperature showed a predictable and significant response. The study by Wellauer et al. (19) clearly demonstrated that hot water immersion (HWI) raised core temperature, while cold water immersion (CWI) significantly decreased it. Even more drastic was the effect on skin temperature, where CWI caused an abrupt decline, confirming a powerful peripheral thermal stimulus.

These thermal alterations triggered equally marked cardiovascular responses. Menzies et al. (20) reported that CWI reduced heart rate (HR) below resting levels, while HWI kept it elevated, indicating increased cardiovascular stress. These changes in HR were directly related to the modulation of peripheral blood flow: CWI induced severe vasoconstriction, drastically reducing flow in the femoral artery, while HWI caused vasodilation, maintaining high flow. The study by Coertjens et al. (23) corroborated this dynamic, showing that CWI accelerated the recovery kinetics of HR, while HWI slowed it down.

Finally, in the metabolic realm, Coertjens et al. (23) provided a key finding: both CWI and HWI significantly increased post-exercise oxygen consumption (EPOC) compared to passive control. This indicates that both interventions pose an additional energy cost to the body during recovery. In the case of CWI, this increase is attributed to the processes of thermogenesis (heat production to combat cold), and in the case of HWI, to the general increase in heat-induced metabolism.

## 4 Discussion

The present systematic review aimed to evaluate the effectiveness of post-exercise water immersion, comparing different temperatures (cold, hot and contrast) on recovery in athletes, analyzing their effects on perceptual, biochemical, performance and physiological parameters. The main findings reveal a complex landscape that challenges the conception of hydrotherapy as a universal recovery strategy. Available evidence indicates that the effects are highly dependent on the water temperature and the specific recovery target. Overall, interventions were not shown to be superior to placebo or a control group in attenuating muscle pain or improving long-term sprint speed recovery. However, specific and divergent effects emerge: hot water immersion proved effective for acute potentiation of anaerobic performance and for improving sleep quality and anabolic hormonal profile, while cold water immersion, although referred to subjectively by athletes, showed a detrimental effect on immediate power performance and induced unique cellular adaptive responses at the chronic level.

### 4.1 Comparison with previous literature

A reiterated finding in the present review is the discrepancy between athletes' subjective perception and the absence of objective evidence demonstrating a superiority of hydrotherapy over placebo or passive control. In relation to late-onset muscle pain (DOMS) and the perception of effort (RPE), the available results are consistent: both the study by Colomer et al. (18) and that by Wellauer et al. (19) showed that the increase in DOMS was comparable in all the groups evaluated, without observing a significant mitigating effect of immersion in cold water (CWI) or hot water (HWI).This aligns with research such as Machado et al. (25), where the results show variable effects: some protocols reduce DOMS (depending on temperature/time), but methodological heterogeneity limits generalization. Similarly, inconsistent results, reported in the literature, as in the study by Moore et al. (26), conclude mixed results for performance and perceptual measures (DOMS, RPE) and highly variable evidence in biomarkers (CK).

Furthermore, a significant finding of this review is the evidence of potential sex-specific responses to water immersion. The corpus of evidence is characterized by a clear male predominance, with 75% of the studies (*n* = 6) focusing exclusively on men. In this context, the study by Wellauer et al. (19) is pivotal, as it observed that HWI resulted in significantly higher CK levels compared to control in a female population—a result that diverges from findings in male cohorts. These differences may be attributed to biological dimorphism, including variations in subcutaneous fat distribution, which influences thermal insulation, and the protective role of estrogen against exercise-induced muscle damage (EIMD). These factors may fundamentally alter the kinetics of heat transfer and inflammatory modulation, suggesting that recovery protocols should be tailored to the biological sex of the athlete.

However, it would be simplistic to dismiss all perceptual benefits. A specific and notable finding came from Horgan et al. (22), who identified that HWI significantly improved sleep quality and reduced fatigue compared to other modalities. This effect, which was not observed with CWI, suggests a distinct mechanism of action, possibly related to the effects of HWI on central thermoregulation, which is known to facilitate sleep onset. Thus, although hydrotherapy does not appear to be a superior tool for the overall management of post-exercise muscle pain, HWI emerges as a specific and promising strategy to optimize key aspects of athlete wellbeing, such as night rest.

The role of the placebo effect remains a critical consideration in the interpretation of hydrotherapy benefits. The methodological inclusion of a credible placebo group in the study by Gustafsson et al. (21) provides a robust framework to distinguish physiological recovery from psychological expectations. Their findings, which showed no objective superiority of CWI or HWI over a placebo for performance return, suggest that the perceived benefits frequently reported by athletes may be largely driven by the “recovery ritual” or the psychological conviction of efficacy. This discrepancy between subjective perception and objective physiological markers underscores the need for future research to incorporate control conditions that account for the psychological influence of recovery interventions.

Analysis of blood markers reveals that the effect of hydrotherapy transcends a simple suppression of inflammation, pointing towards a complex modulation of cellular signaling pathways that depends on the temperature and chronicity of the stimulus. Regarding acute muscle damage (CK), the evidence from this review is conflicting. While Horgan et al. (17) reported a reduction in CK with all immersion modalities, aligning with previous meta-analyses, Wellauer et al. (19), found no such benefit, and even observed an increase in CK with HWI in a female population. This discrepancy underscores the heterogeneity of the response, possibly influenced by the participant's sex or type of exercise. However, when examining chronic effects, Horgan et al. (24) found a clearer result: repeated use of both CWI and HWI reduced baseline CK levels, suggesting a protective or recovery enhancing effect in the long term.

Beyond muscle damage, findings on cytokines and other signaling proteins challenge the paradigm that CWI is purely “anti-inflammatory.” In fact, the most significant finding from the chronic study by Horgan et al. (24) was that repeated CWI increased levels of Heat Shock Protein (HSP-72). This is an indicator of a cellular stress response, positioning CWI not only as a recovery modality, but also as an adaptive stimulus. This result aligns with the literature that warns that CWI can attenuate anabolic signals necessary for muscle hypertrophy, possibly through modulation of these stress pathways. In contrast, chronic HWI appeared to promote a more proadaptive environment, evidenced by reduced anti-inflammatory cytokine IL-1ra and increased growth factors.

### 4.2 Strengths and limitations

A major strength of this review is its currency, incorporating high-quality trials from 2024 to 2025 that challenge previous recovery paradigms. However, several limitations must be considered to contextualize these findings. First, the high methodological heterogeneity in water temperatures and immersion durations prevented a quantitative meta-analysis. Second, the clear predominance of male participants limits the generalizability of the results to elite female athletes, who may exhibit different thermoregulatory and inflammatory responses.

Most importantly, it must be acknowledged that a substantial proportion of the evidence analyzed (50%; *n* = 4) originates from a single research group [Horgan et al.]. While these studies maintained high methodological standards, as evidenced by their PEDro scores (6–7), such a concentration of data may introduce a “laboratory-specific bias.” The consistency in their findings (particularly regarding sleep quality and anabolic hormonal profiles in volleyball and rugby players) might reflect specific laboratory protocols or the characteristics of their particular sub-population rather than a universal phenomenon. Therefore, independent replication by diverse research teams across different athletic disciplines is necessary to strengthen the external validity and global applicability of these conclusions.

### 4.3 Practical applications

Based on the synthesized evidence, practitioners and coaches should adopt a context-specific approach to hydrotherapy:

Prioritize HWI (38–42 °C) when the primary goal is to improve sleep quality, reduce perceived fatigue, or provide an acute potentiating effect before anaerobic performance (e.g., between tournament rounds on the same day).

Exercise caution with CWI (8–15 °C) if explosive power or vertical jump height is required immediately following the intervention, as it may cause an acute decrement in performance.

“Align CWI use with cellular adaptation goals”: Repeated CWI may be used to induce cellular stress responses (HSP-72), but its potential to attenuate anabolic signaling should be considered during hypertrophy-focused training phases.

### 4.4 Future research directions

Future studies should prioritize “placebo-controlled” designs to further isolate the physiological effects of water immersion from psychological expectations. There is a critical need for research focused exclusively on elite female athletes to address the current biological sex gap in the literature. Additionally, longitudinal studies (exceeding 15 weeks) are required to determine how chronic use of HWI affects long-term training adaptations and hormonal health, moving beyond acute recovery metrics.

## 5 Conclusions

This systematic review strongly demonstrates that post-exercise hydrotherapy is not a monolithic or universally beneficial recovery strategy. Its effectiveness is highly dependent on the context, the water temperature, the time of application, and the desired physiological or performance outcome. The evidence analyzed challenges the widespread practice of using cold water immersion (CWI) as the primary method for accelerating objective recovery, as it does not demonstrate consistent superiority over placebo or passive control for attenuating muscle pain or improving performance in the days following exercise.

Moreover, the findings reveal clearly divergent and specific action profiles for each temperature. Cold water immersion (CWI), although often subjectively preferred by athletes, evidences a detrimental acute effect on immediate anaerobic potency. Its main role at the chronic level seems to be that of a cellular stress stimulus, as evidenced by the increase in Heat Shock Protein (HSP-72), positioning it more as a possible adaptive modulation tool than as a simple recovery accelerator.

In contrast, hot water immersion (HWI) emerges with a well-defined benefit profile: it is an effective strategy for acute enhancement of anaerobic performance, significantly improves perceived sleep quality, and promotes a more anabolic hormonal environment through increased testosterone levels. These effects position HWI not only as a recovery modality, but also as a possible adjuvant in training adaptation processes.

The choice between CWI and HWI should not be arbitrary, but a strategic decision: HWI should be considered to enhance performance between short efforts or to improve rest, while CWI should be used with caution, especially if immediate power performance is a priority. Ultimately, the question for the clinician and trainer should not be “should we use hydrotherapy?”, but rather “what specific physiological response do we want to modulate and at what point in the training cycle?”, using water temperature as a precise tool to achieve this.

## Data Availability

The original contributions presented in the study are included in the article/Supplementary Material, further inquiries can be directed to the corresponding author.

## Appendix A

Note on Search Strategy: The syntax of search strings varies across databases (Scopus, PubMed, WOS, etc.) due to differences in indexing systems and Boolean logic. We ensured that the search terms were conceptually identical across all platforms. In Scopus, specific document type filters (excluding “notes”, “editorials”, or “reviews”) were utilized to refine the results according to our primary research inclusion criteria, ensuring a high-quality retrieval process consistent with PRISMA guidelines.

**Table:**

SCOPUS	Results
(TITLE-ABS-KEY (hydrotherap * OR “water immersion” OR “cold water immersion” OR “hot water immersion” OR “contrast water therapy *” OR “thermoneutral water immersion” OR “heat therapy *” OR “cold therapy *” OR cryotherap * OR “thermal therapy *” OR “aquatic therapy *”)) AND (TITLE-ABS-KEY (“muscle fatigue” OR “Exercise-induced Muscle Damage” OR “EIMD” OR exhaustion OR “Exercise Performance” OR “Delayed onset muscle soreness” OR “DOMS” OR “training stress” OR “muscle ache” OR “muscle soreness”)) AND pubyear > 2014 AND pubyear < 2026 AND (EXCLUDE (DOCTYPE, “re”) OR EXCLUDE (DOCTYPE, “no”) OR EXCLUDE (DOCTYPE, “cp”) OR EXCLUDE (DOCTYPE, “sh”) OR EXCLUDE (DOCTYPE, “ch”) OR EXCLUDE (DOCTYPE, “le”) OR EXCLUDE (DOCTYPE, “er”) OR EXCLUDE (DOCTYPE, “ed)”)	292
SPORTDISCUSS	
(TI (hydrotherap * OR “water immersion” OR “cold water immersion” OR “hot water immersion” OR “contrast water therapy” OR “thermoneutral water immersion” OR “heat therapy *” OR “cold therapy” OR “cryotherap * OR” thermal therapy * “OR” aquatic therapy * “) OR AB (hydrotherap * OR” water immersion “OR” cold water immersion “OR” hot water immersion “OR” contrast water therapy * “OR” thermoneutral water immersion “OR” heat therapy * “OR” cold therapy * “OR” cryotherap * OR “thermal therapy *” OR “aquatic therapy *”)) AND (TI (“muscle fatigue” OR “Exercise-induced Muscle Damage” OR “EIMD” OR exhaustion OR “Exercise Performance” OR “Delayed onset muscle soreness” OR “DOMS” OR “stress training” OR “muscle ache” OR “muscle soreness”) OR AB (“muscle fatigue” OR “Exercise-induced Muscle Damage” OR “EIMD” OR exhaustion OR “Exercise Performance” OR “Delayed onset muscle soreness” OR “DOMS” stress training “OR” OR “muscle soreness”)	135
TAYLOR AND FRANCYS	
(title: (hydrotherap * OR “water immersion” OR “cold water immersion” OR cryotherap *) OR abstract: (hydrotherap * OR “water immersion” OR “cold water immersion” OR “hot water immersion” OR “contrast water therapy *” OR “thermoneutral water immersion” OR “heat therapy *” OR “cold therapy *” OR cryotherap * OR “thermal therapy *” OR “aquatic therapy *”)) AND (title: (“muscle fatigue” OR “muscle soreness” OR “DOMS”) OR abstract: (“muscle fatigue” OR “Exercise-induced Muscle Damage” OR “EIMD” OR exhaustion OR “Exercise Performance” OR “Delayed onset muscle soreness” OR “DOMS” OR “training stress” OR “muscle ache” OR “muscle soreness”))	10
WOS	
(TS = (hydrotherap * OR “water immersion” OR “cold water immersion” OR “hot water immersion” OR “contrast water therapy *” OR “thermoneutral water immersion” OR “heat therapy *” OR “cold therapy *” OR cryotherap * OR “thermal therapy *” OR “aquatic therapy *”)) AND (TS = (“muscle fatigue” OR “Exercise-induced Muscle Damage” OR “EIMD” OR exhaustion OR “Exercise Performance” OR “Delayed onset muscle soreness” OR “DOMS” OR “training stress” OR “muscle ache” OR “muscle soreness”))	353
ScienceDirect	
(hydrotherap OR “contrast water” OR cryotherap) AND (“muscle fatigue” OR “DOMS” OR “muscle soreness” OR “muscle ache” OR “training stress”)	74
PubMed	
(((“Hydrotherapy”[Mesh]) OR “Cryotherapy”[Mesh] OR “Hot Temperature”[Mesh] OR “Cold Temperature”[Mesh]) OR (hydrotherap *[tiab] OR “water immersion”[tiab] OR “cold water immersion”[tiab] OR “hot water immersion”[tiab] OR “contrast water therapy *”[tiab] OR “thermoneutral water immersion” [tiab] OR “heat therapy *” [tiab] OR “cold therapy *” “[tiab] OR cryotherap * [tiab] OR” thermal therapy * “‘ [tiab] OR’ aquatic therapy *’ [tiab]) AND (((“Muscle Fatigue”[Mesh]) OR “Myalgia”[Mesh] OR “Athletic Performance” [Mesh]) OR (“muscle fatigue” [tiab] OR “Exercise-induced Muscle Damage”[tiab] OR “EIMD” [tiab] OR exhaustion [tiab] OR “Exercise Performance” [tiab] OR “Delayed onset muscle soreness” [tiab] OR “DOMS”[tiab] OR “stress training” [tiab] OR “muscle acheab” [tiabeness]))	194
TOTAL	1,058
